# Knowledge, Attitudes, and Practices Regarding “New Normal” Guidelines and Quality of Life Among Thai People During the COVID-19 Outbreak: An Online Cross-Sectional Survey

**DOI:** 10.3389/fpubh.2022.914417

**Published:** 2022-07-07

**Authors:** Pathavee Waewwab, Wirichada Pan-ngum, Sukhontha Siri, Bhophkrit Bhopdhornangkul, Wiriya Mahikul

**Affiliations:** ^1^Division of Communicable Disease Control, Rayong Provincial Public Health Office, Rayong, Thailand; ^2^Mahidol-Oxford Tropical Medicine Research Unit, Faculty of Tropical Medicine, Mahidol University, Bangkok, Thailand; ^3^Department of Tropical Hygiene, Faculty of Tropical Medicine, Mahidol University, Bangkok, Thailand; ^4^Department of Epidemiology, Faculty of Public Health, Mahidol University, Bangkok, Thailand; ^5^Infectious of Disease Control and Entomology Section, Division of Health Promotion and Preventive Medicine, Royal Thai Army Medical Crops, Bangkok, Thailand; ^6^Princess Srisavangavadhana College of Medicine, Chulabhorn Royal Academy, Bangkok, Thailand

**Keywords:** coronavirus disease 2019 (COVID-19), knowledge, attitudes, practices, “new normal” guidelines, quality of life

## Abstract

In Thailand, strict prevention and control strategies have been implemented to mitigate the rapid spread of coronavirus disease 2019 (COVID-19). “New normal” guidelines and a series of mobile health applications have been introduced by the healthcare sector and implemented to aid the disease control monitoring and prevention of widespread outbreaks. This study aimed to assess the knowledge, attitudes, and practices (KAP) regarding “new normal” guidelines and quality of life (QOL) among Thai people during the COVID-19 outbreak, and to determine the association between KA, QOL, and practices. An online cross-sectional survey was conducted from 7 June to 12 September 2021 among Thai people in Public Health Region 6 aged ≥ 18 years old. Of the 506 survey participants, 80.3% were female, and 65.0% were 25–59 years old. The survey revealed that 52.2% of participants were classified as having more accurate knowledge, 58.9% were classified as having more positive attitudes, and 80.8% were classified as having more frequent practices regarding “new normal” guidelines, and 54.7% had high QOL. Of the participants, 93.7% agreed that “people who have been fully vaccinated should wear a mask while outside,” and 95.5% wore a face mask outdoors in crowded places. However, 60.9% of participants misunderstood some details regarding online applications for contact tracing and vaccination services, 44.2% felt that these applications were difficult to use, and 33.4% rarely or never downloaded or used these applications. In logistic regression analyses, accurate knowledge of COVID-19 was associated with higher education, being a government employee, monthly family income > 30,000 Thai Baht, and regular use of social media. More positive attitudes regarding “new normal” guidelines and high QOL were associated with positive practices. High QOL was associated with older age, and higher education. Enhancement of attitudes and QOL is also important for improving practices in the general population during the COVID-19 pandemic. Significant factors identified in KAP will be crucial for developing effective prevention and control programs to mitigate the spread of COVID-19. To implement mobile health applications effectively, more work is required to improve the ease of use and promotion strategies.

## Introduction

Coronavirus disease 2019 (COVID-19) is a highly infectious respiratory disease, which was first recorded in Wuhan City, People's Republic of China, in December 2019. The pandemic is widespread, covering 221 countries. The World Health Organization (WHO) reported that there were more than 227 million confirmed cases of COVID-19 and 4.6 million deaths, as of 16 September 2021 (1). In Thailand, 1.4 million confirmed cases and 14,953 deaths were reported, as of 16 September 2021 (2).

Severe acute respiratory syndrome coronavirus 2 (SARS-CoV-2) can be transmitted from person to person by droplet nuclei and *via* airborne mechanisms (3, 4), as well as being released in feces (5, 6). Moreover, rubbing the eyes (infection through conjunctiva) or touching the face and mouth may also be COVID-19 infection pathways (5). The signs and symptoms of COVID-19 include fever, cough, sneezing, runny nose, wheezing, and sore throat, among others. In severe cases, patients may get COVID-19 with pneumonia. In Thailand, the first confirmed case was reported in January 2020, and mass clusters, such as outbreaks among crowds at Thai boxing stadiums, entertainment venues, and people returning from the dawah pilgrimage, were reported in March 2020 (7). The Thai government enacted policy to establish a Center for Administrative Situation of the Coronavirus Disease 2019 and implemented various measures, such as appropriate mask wearing, hand washing, social distancing, and reducing gathering sizes to prevent and control the spread of COVID-19 within Thailand. Thailand's Center for COVID-19 Situation Administration (CCSA) announced the declaration of a state of emergency, travel restrictions, and curfew hours, which were imposed to prevent the spread of infections, from 10 p.m.−4 a.m. (8, 9). Moreover, closing high-risk locations was found to effectively mitigate the transmission of the first wave of COVID-19 (10). However, these policies also have the potential to impact socioeconomic status, quality of life (QOL), and lifestyles of Thai people, which was changed to a so-called “new normal” state (11).

“New normal” guidelines refer to the requirement for Thai people to modify their behaviors and change their lifestyle patterns to prevent COVID-19 infection (11–13). Examples of such changes include working from home, covering the mouth when coughing and sneezing, wearing a cloth mask or surgical mask, maintaining a distance of at least 1 m from others, washing hands frequently, checking body temperature, self-assessment and tracing using the MorChana application (Ministry of Digital Economy and Society, Bangkok, Thailand) to track movements (14), checking in and checking out of visited locations using ThaiChana [“Thais win” in Thai language; Center for COVID-19 Situation Administration (CCSA), Bangkok, Thailand] (15), accessing vaccine services with Morprom (Ministry of Public Health, Bangkok, Thailand) (16), and businesses and services providing online instead of offline services to adapt to the current situation. Lifestyle changes to shift to a “new normal” mode may therefore be essential measures for the prevention of subsequent waves of COVID-19 and re-emergence in future outbreaks. Moreover, the Thai government has provided COVID-19 vaccinations to reduce the severity and spread of the disease. As of 16 September 2021, the coverage of COVID-19 vaccination in Thailand had reached 45,126,911 doses (2).

However, changes in behavior regarding “new normal” guidelines might change when the COVID-19 situation in the country is under control. Lifting more stringent measures might affect attitudes and practices, leading to a decrease in the effectiveness of controlling the spread of COVID-19 in the Thai population (17). The survey by the Thai Ministry of Public Health and Bureau of International Health Policy Program (IHPP) reported trends toward decreases in non-pharmaceutical interventions when measures were relaxed, such as reductions in the use of surgical masks, pre-cooked meals, and use of mobile health applications (18). Previous research done during the SARS pandemic in 2003 showed that peoples' knowledge and attitudes related to SARS were related to public nervousness and panic. A decrease in public panic might reduce the knowledge, attitudes, and practices (KAP) required to prevent further spread of infectious and emerging diseases (19, 20).

Several survey studies have been conducted to assess the KAP and QOL toward COVID-19 such as a study among people living near the Northern Thai border. This study revealed that preparedness to respond to COVID-19 was higher among educated young women than that among older men with no formal education (21). More than 70% of participants in that study lacked knowledge about prevention (21). A previous study conducted in 22 countries reported that females, individuals with a higher level of education, and urban residents had significantly higher knowledge and practice scores, and correlations between all KAP components (22). One previous study reported that people with higher levels of knowledge and behavior for preventing COVID-19 had lower scores regarding practices (23). In China, a study of industrial workers revealed that more than 30% of workers had a low level of practice regarding measuring body temperature daily, wearing masks while working, and physical distancing (24). Several previous studies reported significant correlations between KAP and QOL (25–27). A study of QOL using the World Health Organization Quality of Life (WHOQOL-BREF) scale among Thai students during the COVID-19 outbreak revealed that moderate QOL was reported for most people (28). Another study revealed that there was a lower mean QOL using the EQ-5D-5L among older adults with diabetes during COVID-19 outbreaks (29) and attitudes were positively associated with QOL (25). Icek Ajzen stated that the more favorable attitude and subjective norm should be the person's intention to perform their behavior (30). Therefore, in the current study, we aimed to assess KAP regarding the “new normal” guidelines and QOL among Thai people during the COVID-19 outbreak, and to determine the association between KA, QOL, and practices regarding “new normal” guidelines.

## Methods

### Participation and Procedure

An online cross-sectional survey during COVID-19 outbreaks was conducted from 7 June to 12 September 2021, involving Thai people in Public Health Region 6 who were 18 years old and above. To estimate the sample size an infinite population proportion formula, *n* = Z^2^p(1-p)/d^2^ was used with estimated knowledge level 85% (22), 3% precision (d), 95% confidence level, and considering 5% non-response proportion. Therefore, the minimum calculated sample size was 573. The response rate was calculated by dividing the number of people returning the online survey by total number of people access to the form online i.e., 506 out of 573 filled and return the questionnaire, thus giving 88% response rate. The original sample size calculated of 573 assuming the non-response rate of 5%. Here the final sample size was slightly smaller than expected due to the high non-response rate of the study. However, this sample size was considered sufficient and adhered with sample size of a previous study among this specific population (24). Stratified random sampling was applied to select the study participants. A self-reported questionnaire was designed using the Google survey tool (Google Forms; Google, California, United States). The generated link was transformed into a quick response (QR) code and shared with all Provincial Public Health Offices in Public Health Region 6 using official government letters. The QR code was shared afterward with the public on social media, such as *via* the website of the Provincial Public Health Offices, as well as *via* Facebook, Line, and Twitter accounts. All participants in this study gave their written informed consent to participate. A total of 506 survey participants met the inclusion criteria (Supplementary Material): (1) Thai nationality, (2) living in Public Health Region 6 (Chonburi, Rayong, Chanthaburi, Trat, Samutprakarn, Chachoengsao, Prachinburi, and Sa Kaeo Provinces, Thailand) for at least 6 months, (3) lower education (below bachelor's degree) as a minimal level of education, (4) having Internet access, and (5) voluntary participation.

### Measures

The online questionnaire included informed consent, questions regarding socio-demographic characteristics, KAP, and QOL.

Socio-demographic variables included age (school-aged [20–24 years], working-aged [25–59 years], and elderly [≥60 years]), gender, marital status, income, education, occupation, and province of residence.

KAP was assessed regarding the “new normal” guidelines, including 15 questions for knowledge, 12 questions for attitudes, and 12 questions for practices. Knowledge questions had possible responses of “Yes,” “No,” and “Don't know.” Correct answers (Yes) were coded as 1 point, whereas wrong answers (No/Don't know) were coded as 0 points. The level of KAP was categorized using modified Bloom's cut off between 75 and 80% to determine more accurate knowledge, more positive attitude, and more frequent practice (31). Total scores for knowledge were classified into two categories: “Less accurate” (scores of 0–11 points), and “More accurate” (scores of 12–15 points). Attitudes were measured using positive and negative questions. Positive questions were measured on a 5-point Likert scale, as follows: “Strongly agree” (5 points), “agree” (4 points), “Neither disagree nor agree” (3 points), “Disagree” (2 points), and “Strongly disagree” (1 point). Negative questions were also measured on a 5-point Likert scale, as follows: “Strongly agree” (1 point), “agree” (2 points), “Neither disagree nor agree” (3 points), “Disagree” (4 points), and “Strongly disagree” (5 points). Total scores for attitudes were classified into two categories: “Less positive” (scores of 12–43 points) and “More positive” (scores of 44–60 points). Practice was measured using positive and negative questions. Positive questions were measured on a 5-point Likert scale, as follows: “Always” (5 points), “Very often” (4 points), “Sometimes” (3 points), “Rarely” (2 points), and “Never” (1 point). Negative questions were also measured on a 5-point Likert scale, as follows: “Always” (1 point), “Very often” (2 points), “Sometimes” (3 points), “Rarely” (4 points), and “Never” (5 points). Total scores for practices were classified into two categories: “Less frequent” (scores of 10–43 points) and “More frequent” (scores of 44–60 points).

QOL regarding “new normal” guidelines was assessed using the Thai version of the WHOQOL-BREF (WHOQOL-BREF-THAI) (32). Previous studies in Thailand used this form to evaluate QOL among health professional students and elderly people (28, 33). The questions included three negative questions and 23 positive questions. Positive questions were measured on a 5-point Likert scale, as follows: “Never” (1 point), “Rarely” (2 points), “Sometimes” (3 points), “Often” (4 points), and “Always” (5 points). Negative questions were also measured on a 5-point Likert scale, as follows: “Never” (5 points), “Rarely” (4 points), “Sometimes” (3 points), “Often” (2 points), and “Always” (1 point). Total QOL scores were classified into two categories: “Low QOL” (scores of 26–95 points) and “High QOL” (scores of 96–130 points). Different domains of the QOL were categorized using the cut off from previous study in Thailand (34). Total QOL scores in the physical health domain were classified into two categories: “Low QOL” (scores of 7–26 points) and “High QOL” (scores of 27–35 points). Total QOL scores in the psychological domain were classified into two categories: “Low QOL” (scores of 6–22 points) and “High QOL” (scores of 23–30 points). Total QOL scores in the social relationships domain were classified into two categories: “Low QOL” (scores of 3–11 points) and “High QOL” (scores of 12–15 points). Total QOL scores in the environment domain were classified into two categories: “Low QOL” (scores of 8–29 points) and “High QOL” (scores of 30–40 points).

To maximize scientific rigor in this research, we adopted the processes in the questionnaire methodologies including translation and back translation, peer and expert review, and piloting. An online questionnaire was drafted and sent to three academic experts including epidemiologist, public health technical officer, and medical doctor who had experience in preventing and controlling COVID-19 outbreaks. Each question was validated using the index of item objective congruence (IOC) with a cutoff value of ≥ 0.5. After content validity was conducted, the questionnaire was pilot tested with 30 individuals to confirm its reliability. The reliability of the questionnaire was analyzed using coefficient analysis. The Cronbach's alpha coefficients for knowledge, attitudes, and practices were 0.52, 0.71, and 0.69, respectively. However, the reliability coefficient of knowledge was low because the questions have various different aspects. While, Cronbach's alpha coefficient for QOL was 0.9.

### Statistical Analysis

The data analysis was performed using Microsoft Excel (Microsoft Corporation, Redmond, WA) for editing, sorting, and coding. The complete Excel file was then imported into SPSS software (version 26, Chicago, IL, USA). Descriptive statistics (such as frequencies, percentages, means, standard deviations) and first-order analyses (such as the Chi-square test) were performed. Pearson's correlation analysis was applied to examine the relationship between respondents' KAP and QOL. For identifying significant associations between categorical dependent and independent variables, binary logistic regression and multiple logistic regression were performed with 95% confidence intervals (CIs).

### Ethical Considerations

The study was conducted in a full-board review of Human Research Ethics Committee Chulabhorn Research Institute. Formal ethics approval was granted on 23 April 2021 (Project code: 025/2564). The objectives and procedures of the study were described in the consent form. Anonymity and confidentially were strictly maintained.

## Results

Of the 506 survey participants, 80.3% were female, with an average age of 39.6 ± 14.7 years (standard deviation [SD]) ranging from 20 to 76 years. Of the participants, 65.0% were in the working-aged group (25–59 years), and 46.2% were unmarried. In addition, 54.5% of participants had a bachelor's degree, and 48.4% worked in the non-government sector. Of the participants, 63.2% had monthly family incomes between 10,000 and 30,000 Thai Baht (THB), 16.6% were faced with job loss, and 68.6% were faced with income loss. In addition, 87.4% of respondents regularly used social media. Of the participants, 24.3% had a chronic disease, 4.9% had ever smoked, and 36.6% had ever drank alcohol. Moreover, 23.3% of participants lived in Chonburi and 89.9% were willing to get vaccinated against COVID-19 (see Table 1).

**Table 1:** Demographic characteristics of participants (*N* = 506).

**Variables**	***n***	**(%)**
Gender		
Male	100	19.8
Female	406	80.2
Age (years)		
20–24	124	24.5
25–59	329	65.0
≥60	53	10.5
Marital status		
Unmarried	234	46.2
Married	222	43.9
Divorced	50	9.9
Education		
Lower education (below bachelor's degree)	177	35.0
Bachelor's degree	276	55.5
Higher education (above bachelor's degree)	53	10.5
Occupation		
Student	103	20.5
Government employee	122	24.1
Non-government employee	245	48.4
Unemployed	36	7.1
Monthly family income		
<10,000 THB	60	11.9
10,000–30,000 THB	320	63.2
> 30,000 THB	126	24.9
Job loss		
Yes	84	16.6
No	422	83.4
Income loss		
Yes	347	68.6
No	159	31.4
Social media use		
Never or sometimes	64	12.6
Every day	442	87.4
Underlying disease		
Yes	123	75.7
No	383	24.3
Smoking		
Yes	25	4.9
No	481	95.1
Drinking alcohol		
Yes	185	36.6
No	321	63.4
Willingness to get vaccinated against COVID-19		
Yes	455	89.9
No	11	2.2
Not sure	40	7.9
Province
Chonburi	118	23.3
Rayong	57	11.3
Chanthaburi	55	10.9
Trat	19	3.8
Samut Prakan	116	22.9
Chachoengsao	75	14.8
Prachin Buri	33	6.5
Sa Kaeo	33	6.5

Pearson's correlation statistics indicated significant positive relationships (*p*-value < 0.01) between knowledge and practices (*r* = 0.142), attitudes and practices (*r* = 0.392), and QOL and practices (*r* = 0.348) (see Table 2).

**Table 2:** Pearson's correlation table for the relationships between knowledge, attitudes, and practices regarding “new normal” guidelines and quality of life among Thai people during the COVID-19 outbreak.

**Variables**	**Knowledge**	**Attitudes**	**Practices**	**Quality of life**
Knowledge	1			
Attitudes	0.195**	1		
Practices	0.142**	0.392**	1	
Quality of life	0.079	0.272**	0.348**	1

**p-value < 0.01.

### Knowledge

For knowledge regarding “new normal” guidelines, participants had an average knowledge score of 11.53 ± 1.84 points (SD), with scores ranging from 2 to 15 points. In addition, 52.2% of participants were classified as having more accurate knowledge. Most respondents possessed accurate knowledge regarding “new normal” guidelines: 97.6% of participants agreed that “People in contact with someone infected with SARS-CoV-2 should be immediately quarantined and tested for the virus,” 96.4% agreed that “Screening of people by measuring body temperature is a simple non-contact COVID-19 screening tool,” 96.2% agreed that “Maintaining a distance of at least 2 meters can help to prevent COVID-19,” and 93.7% agreed that “People who have been fully vaccinated should wear a mask while outside” (Table 3). However, 60.9% of participants misunderstood the use of the MorChana application for vaccination services. This application is used for contact tracing. Moreover, 44.1% of participants did not know that the ThaiChana application notifies users about locations of COVID-19 cases *via* short message service (SMS) if they scan the QR code when they arrive and leave the premises.

**Table 3:** Responses to the questionnaire about knowledge regarding “new normal” guidelines during the COVID-19 outbreak.

**Statement**	***N*** **(%)**	
	**Correct answer**	**Incorrect answer**
1. Wearing a cloth mask can prevent COVID-19 more effectively than a surgical mask.	368 (72.7)	138 (27.3)
2. People can reuse masks.	373 (93.7)	32 (6.3)
3. Cooking food at a temperature of > 70 °C for at least 2 min destroys the coronavirus.	279 (55.1)	227 (44.9)
4. Normal saline can be used as a disinfectant for COVID-19.	329 (65.0)	177 (35.0)
5. Maintaining a distance of at least 2 meters can prevent COVID-19.	487 (96.2)	19 (3.8)
6. Using hand sanitizer containing at least 60% alcohol for at least 20 s can inactivate the SARS-CoV-2 virus.	463 (91.5)	43 (8.5)
7. Washing with soap and water for at least 20 s can inactivate the SARS-CoV-2 virus.	464 (91.7)	42 (8.3)
8. People in contact with someone infected with SARS-CoV-2 should be immediately quarantined for a general observation period of 7 days.	360 (71.1)	146 (28.9)
9. People in contact with someone infected with SARS-CoV-2 should be immediately quarantined and tested for the virus.	494 (97.6)	12 (2.4)
10. The MorChana application is used for vaccination services.	198 (39.1)	308 (60.9)
11. The ThaiChana (Thais win) application notifies users if they have visited places with active COVID-19 cases *via* SMS messages.	223 (44.1)	283 (55.9)
12. Screening of people by measuring body temperature is a simple non-contact COVID-19 screening tool.	488 (96.4)	18 (3.6)
13. Measuring body temperature from the hand is less accurate than measuring body temperature from the head.	301 (59.5)	205 (40.5)
14. The COVID-19 vaccine is 100% effective.	432 (85.4)	74 (14.6)
15. People who have been fully vaccinated should wear a mask while outside.	474 (93.7)	32 (6.3)

### Attitudes

The results for attitudes regarding “new normal” guidelines revealed that participants had average attitude scores of 45.33 ± 7.05 points (SD), with scores ranging from 24–60 points. 60% of participants had more positive attitude scores ranging from 44–60 points. In response to the positive attitude questions, 24.1% of participants strongly agreed that “Wearing a cloth mask could prevent COVID-19 transmission,” whereas 25.3% responded “neither disagree nor agree” or “disagree” (Table 4). Of the participants, 71.0% agreed or strongly agreed with the statements “It's important to maintain a distance of at least 2 meters to prevent the spread of COVID-19” and “Quarantining for 14 days is a suitable duration.” In addition, 26.7% of respondents reported that “It's a waste of time to scan the ThaiChana QR code.” In response to the negative attitude questions, 44.2% of participants agreed or strongly agreed that “Using an application for vaccine service (Morprom) is difficult,” 13.9% agreed that “Wearing a surgical mask or cloth mask is difficult,” 6.6% agreed that “Pre-cooking food can cause changes in the flavor and make it less delicious,” 8.7% agreed that “It's a waste of time to clean a surface with alcohol 70%,” 27.6% agreed that “It's a waste of time to scan the ThaiChana QR code,” and 13.0% agreed that “It's a waste of time to measure body temperature before entering crowded places.” Moreover, 10.4% of participants agreed or strongly agreed that “Vaccination against COVID-19 has a greater negative than positive impact.”

**Table 4:** Responses to the questionnaire of attitudes regarding “new normal” guidelines during the COVID-19 outbreak.

**Statement**	***N*** **(%)**				
	**Strongly agree**	**Agree**	**Neutral**	**Disagree**	**Strongly disagree**
1.Wearing a cloth mask can prevent COVID-19 transmission	122 (24.1)	100 (19.8)	156 (30.8)	83 (16.4)	45 (8.9)
2. Wearing a surgical mask or cloth mask is difficult.	21 (4.2)	49 (9.7)	105 (20.7)	126 (24.9)	205 (40.5)
3. Pre-cooking food can cause changes in the flavor and make it less delicious	13 (2.6)	20 (4.0)	66 (13.0)	108 (21.3)	299 (59.1)
4. It's a waste of time to clean a surface with 70% alcohol	17 (3.4)	27 (5.3)	78 (15.4)	116 (22.9)	268 (53.0)
5. It's important to maintain a distance of at least 2 meters to prevent the spread of COVID-19	282 (55.7)	55.7 (26.9)	58 (11.5)	22 (4.3)	8 (1.6)
6. It's a waste of time to wash hands using alcohol gel or soap, and to clean with water before touching the face	22 (4.4)	27 (5.3)	84 (16.6)	119 (23.5)	254 (50.2)
7. Quarantining for 14 days is a suitable duration	212 (41.9)	150 (29.7)	86 (17.0)	29 (5.7)	29 (5.7)
8. It's not important to use the MorChana application.	126 (24.9)	99 (19.5)	146 (28.9)	80 (15.8)	55 (10.9)
9. It's a waste of time to scan the ThaiChana QR code.	70 (13.8)	70 (13.8)	145 (28.7)	109 (21.6)	112 (22.1)
10. It's a waste of time to measure body temperature before entering crowded places	30 (5.9)	36 (7.1)	96 (19.0)	124 (24.5)	220 (43.5)
11. Using the application for vaccine service (Morprom) is difficult.	148 (29.2)	76 (15.0)	113 (22.3)	68 (13.5)	101 (20.0)
12. Vaccination against COVID-19 has a greater negative than positive impact	30 (5.9)	23 (4.5)	96 (19.0)	133 (26.3)	224 (44.3)

### Practices

For practices regarding “new normal” guidelines, participants had an average score of 49.15 ± 6.09 points (SD), ranging from 33 to 60 points. In addition, 80.8% of participants were classified as performing more frequent practices, with scores ranging from 44 to 60 points. In response to the positive practice questions, 95.5% of participants always wore masks when going to public places, 85.0% always measured their body temperature before entering crowded places, and 83.6% very often or always maintained a distance of at least 2 meters to prevent the spread of COVID-19 (Table 5). In addition, 80.0% of participants very often or always washed their hands using alcohol gel or soap and cleaned them with water before touching their faces. Moreover, 64.6% of participants regularly checked up-to-date information about COVID-19 vaccination The negative practice questions revealed that 5.0% of participants very often or always ate raw food and 6.6% of participants visited people in crowded places.

**Table 5:** Responses to the questionnaire about practices regarding “new normal” guidelines during the COVID-19 outbreak.

**Statement**	**Percentage**				
	**Always**	**Usually**	**Sometimes**	**Seldom**	**Never**
1. Wearing masks when you go to public places	95.5	3.2	1.0	0	0.3
2. Eating raw food	2.2	2.8	23.8	42.1	29.1
3. Cleaning surfaces with 70% alcohol	36.8	29.2	26.3	5.9	1.8
4. Maintaining a distance of at least 2 meters to prevent the spread of COVID-19	51.0	32.6	13.8	1.6	1.0
5. Meeting people in crowded places	3.8	2.8	18.0	41.8	33.6
6. Washing hands using alcohol gel or soap and cleaning with water before touching the face	55.1	24.9	16.8	2.2	1.0
7. Quarantining for 14 days after being in contact with COVID-19-positive or high risk people	53.8	14.8	10.7	5.7	15.0
8. Using MorChana application.	35.6	12.5	18.5	17.6	15.8
9. Using and scanning ThaiChana QR codes.	31.8	17.6	27.3	15.0	8.3
10. Measuring body temperature before entering crowded places	85.0	10.7	3.6	0.7	0
11. Using application for vaccine service (Morprom).	46.8	16.2	14.4	11.1	11.5
12. Keeping updated about information regarding COVID-19 vaccines.	64.6	22.9	9.5	1.8	1.2

### Quality of Life

For QOL during the COVID-19 outbreak, participants had an average QOL score of 96.97 ± 14.18 points (SD), with scores ranging from 42 to 129 points, and 54.7% of participants had a high QOL score (ranging from 96 to 130 points). In the QOL domains, 56.0% of participants had a good QOL score for physical health, 58.1% had a good QOL score for psychological health, and 64.0% had a good QOL score for social relationships, while 54.0% of participants had a moderate QOL score in the environmental domain. In addition, 20.5% of participants reported “Having negative feelings such as loneliness, depression, and anxiety during the COVID-19 outbreak,” and 12.9% experienced “Dissatisfaction with the accessibility of health services” (Table 6). Of the participants, 17.2% experienced “Dissatisfaction with financial resources.”

**Table 6:** Responses to the questionnaire of quality of life statements regarding “new normal” guidelines during the COVID-19 outbreak.

**Statement**	**Percentage**				
	**An extreme amount**	**Very much**	**A moderate amount**	**A little**	**Not at all**
1. Having good health perception	25.1	37.9	33.0	2.2	1.8
2. Having pain	9.1	21.7	37.8	22.1	9.3
3. Having good physical functionality	31.6	42.1	23.5	2.4	0.4
4. Having good sleep	24.7	36.0	31.6	5.7	2.0
5. Having positive feelings	22.5	38.3	30.9	6.5	1.8
6. Having concentration	18.0	45.5	31.4	4.5	0.6
7. Having self-esteem	27.1	44.4	23.5	4.0	1.0
8. Accepting body image	33.6	44.6	20.0	1.6	0.2
9. Having negative feelings	5.1	15.4	31.9	31.2	16.4
10. Having good ability to perform daily activities	23.7	47.6	24.9	3.0	0.8
11. Dependence on medication	30.6	28.5	26.3	10.1	4.5
12. Satisfaction with working capacity	22.1	44.5	27.5	5.1	0.8
13. Having good personal relationships	25.1	46.8	23.9	3.4	0.8
14. Having good social support	26.7	46.7	21.1	4.7	0.8
15. Having physical safety and security	17.0	37.8	33.4	7.1	4.7
16. Satisfaction with home environment	29.1	43.5	20.9	4.9	1.6
17. Satisfaction with financial resources	12.3	23.7	46.8	14.0	3.2
18. Satisfaction with access to health services	19.8	34.3	33.0	10.5	2.4
19. Satisfaction with information	22.1	49.0	26.1	2.2	0.6
20. Having leisure time	14.6	30.4	40.4	13.0	1.6
21. Having a good physical environment	22.9	38.1	33.7	4.7	0.6
22. Satisfaction with transportation	13.0	28.9	42.3	11.3	4.5
23. Having good spirituality	36.6	41.2	17.8	3.6	0.8
24. Having good self-mobility	34.6	38.9	21.2	4.7	0.6
25. Satisfaction with sex	22.5	31.6	30.9	8.1	6.9
26. Your level of QOL	18.2	40.5	35.4	5.1	0.8

### Factors Related to KAP Regarding “New Normal” Guidelines

For knowledge regarding the “new normal” guidelines, the association analysis revealed that participants with more accurate knowledge were significantly more likely to be (i) participants (69.8%) with a higher level of education (above bachelor's degree) compared with those (38.4%) with a lower level of education (below bachelor's degree) (*p* < 0.001) (see Table 7), (ii)participants (69.7%) who worked in a government office compared with those (47.2%) who were unemployed (*p* < 0.001), (iii) participants (69.0%) with a monthly family income higher than 30,000 THB compared with those with a monthly family income lower than 10,000 THB (43.3%) and 10,000–30,000 THB (47.2%) (*p* < 0.001), (iv) participants (54.1%) who used social media every day compared with those (39.1%) who never or sometimes used social media (*p* = 0.026).

**Table 7:** Factors related to knowledge, attitudes, and practices regarding “new normal” guidelines among Thai people during the COVID-19 outbreak.

**Variables**	**Knowledge regarding “new normal” guidelines**								**Attitudes regarding “new normal” guidelines**								**Practices regarding “new normal” guidelines**							
	**Less accurate**		**More accurate**		**x^**2**^**	**OR**	**95% CI**	***p-value***	**Less accurate**		**More accurate**		**x^**2**^**	**OR**	**95% CI**	***p-value***	**Less accurate**		**More accurate**		**x^**2**^**	**OR**	**95% CI**	***p-value***
	***N***	**%**	***N***	**%**	***(p-value)***				***N***	**%**	***N***	**%**	***(p-value)***				***N***	**%**	***N***	**%**	***(p-value)***			
Gender																								
Male	49	49.0	51	51.0	0.069	0.943	0.609–1.461	0.793	46	46.0	54	54.0	1.233	0.779	0.502–1.211	0.268	20	20.0	80	80.0	0.055	0.936	0.541–1.621	0.814
Female	193	47.5	213	52.5	(0.793)	Ref.			162	39.9	244	60.1	(0.268)	Ref.			77	19.0	329	81.0	(0.814)	Ref.		
Age (years)																								
20–24	54	43.5	70	56.5	2.551	1.691	0.884–3.235	0.113	70	56.5	54	43.5	16.042	**0.468**	**0.242–0.904**	**0.024**	32	25.8	92	74.2	5.245	0.438	0.179–1.067	0.069
25–59	158	48.0	171	52.0	(0.279)	1.412	0.787–2.533	0.248	118	35.9	211	64.1	**(<0.001)**	1.084	0.595–1.973	0.793	58	17.6	271	82.4	(0.073)	0.711	0.306–1.654	0.428
≥ 60	30	56.6	23	43.4		Ref.			20	37.7	33	62.3		Ref.			7	13.2	46	86.8		Ref.		
Marital status																								
Unmarried	111	47.4	123	52.6	0.027	1.023	0.555–1.885	0.942	112	47.9	122	52.1	8.251	0.561	0.296–1.063	0.076	56	23.9	178	76.1	6.708	0.698	0.319–1.524	0.367
Married	107	48.2	115	51.8	(0.986)	0.992	0.537–1.833	0.980	79	35.6	143	64.4	**(0.016)**	0.932	0.489–1.780	0.832	32	14.4	190	85.6	**(0.035)**	1.303	0.578–2.938	0.523
Divorced	24	48.0	26	52.0		Ref.			17	34.0	33	66.0		Ref.			9	18.0	41	82.0		Ref.		
Education level																								
Lower education (below bachelor's degree)	109	61.6	68	38.4	23.297	**0.270**	**0.139–0.522**	**<0.001**	65	36.7	112	63.3	2.161	1.321	0.708–2.464	0.381	27	15.3	150	84.7	2.770	1.626	0.758–3.486	0.212
Bachelor's degree	117	42.4	159	57.6	**(<0.001)**	0.588	0.312–1.107	0.100	120	43.5	156	56.5	(0.339)	0.997	0.551–1.803	0.991	58	21.0	218	79.0	(0.250)	1.100	0.543–2.227	0.791
Higher education (above bachelor's degree)	16	30.2	37	69.8		Ref.			23	43.4	30	56.6		Ref.			12	22.6	41	77.4		Ref.		
Occupation																								
Student	44	42.7	59	57.3	27.483	0.811	0.402–1.635	0.558	58	56.3	45	43.7	18.005	**0.439**	**0.200–0.960**	**0.039**	27	26.2	76	73.8	6.187	0.679	0.267–1.731	0.418
Government employee	37	30.3	85	69.7	**(<0.001)**	**2.568**	**1.201–5.489**	**0.015**	35	28.7	87	71.3	**(<0.001)**	1.405	0.561-3.081	0.496	16	13.1	106	86.9	(0.103)	1.599	0.601–4.255	0.347
Non-government employee	142	58.0	103	42.0		1.499	0.700–3.211	0.298	102	41.6	143	58.4		0.792	0.383–1.638	0.530	47	19.2	198	80.8		1.017	0.420–2.463	0.970
Unemployed	19	52.8	17	47.2		Ref.			13	36.1	23	63.9		Ref.			7	19.4	29	80.6		Ref.		
Monthly family income																								
<10,000 THB	34	56.7	26	43.3	19.445	**0.343**	**0.182–0.647**	**0.001**	30	50.0	30	50.0	4.030	0.537	0.287–1.002	0.051	9	15.0	51	85.0	1.987	1.694	0.745–3.851	0.208
10,000–30,000 THB	169	52.8	151	47.2	**(<0.001)**	**0.401**	**0.259–0.620**	**<0.001**	134	41.9	186	58.1	(0.133)	0.745	0.485–1.143	0.178	59	18.4	261	81.6	(0.370)	1.323	0.801–2.185	0.275
>30,000 THB	39	31.0	87	69.0		Ref.			44	34.9	82	65.1		Ref.			29	23.0	97	77.0		Ref.		
Social media use																								
Never or Sometimes	39	60.9	25	39.1	5.048	**0.544**	**0.319–0.930**	**0.026**	33	51.6	31	48.4	3.309	0.616	0.364–1.042	0.071	9	14.1	55	85.9	1.233	1.519	0.723–3.192	0.270
Every day	203	45.9	239	54.1	**(0.026)**	Ref.			175	39.6	267	60.4	(0.071)	Ref.			88	19.9	354	80.1	(0.270)	Ref.		
Underlying disease																								
Yes	55	44.7	68	55.3	0.630	1.180	0.784–1.774	0.428	49	39.8	74	60.2	0.108	1.072	0.708–1.622	0.742	23	18.7	100	81.3	0.023	1.041	0.619–1.750	0.879
No	187	48.8	189	52.6	(0.428)	Ref.			159	41.5	224	58.5	(0.742)	Ref.			74	19.3	309	80.7	(0.879)	Ref.		
Smoking																								
Yes	13	52.0	12	48.0	0.184	0.839	0.375–1.876	0.669	14	56.0	11	44.0	2.410	0.531	0.236–1.194	0.126	6	24.0	18	76.0	0.396	0.739	0.287–1.902	0.531
No	229	47.6	252	52.4	(0.669)	Ref.			194	40.3	287	59.7	(0.126)	Ref.			91	18.9	390	81.1	(0.531)	Ref.		
Drinking alcohol																								
Yes	98	53.0	87	47.0	3.096	0.722	0.502–1.038	0.079	91	49.2	94	50.8	7.869	**0.592**	**0.410–0.855**	**0.005**	39	21.1	146	78.9	0.687	0.826	0.525–1.299	0.407
No	144	44.9	177	55.1	(0.079)	Ref.			117	36.4	204	63.6	**(0.005)**	Ref.			58	18.1	263	81.9	(0.407)	Ref.		
Willingness to get vaccinated against COVID-19																								
Yes	218	47.9	237	52.1	0.028	0.984	0.515–1.879	0.960	181	39.8	274	60.2	3.879	1.514	0.792–2.893	0.210	83	18.2	372	81.8	2.849	1.494	0.703–3.176	0.297
No	5	45.5	6	54.5	(0.986)	1.086	0.284–4.143	0.904	7	63.6	4	36.4	0.144	0.571	0.144–2.262	0.425	4	36.4	7	63.6	(0.241)	0.583	0.141–2.418	0.457
Not sure	19	47.5	21	52.5		Ref.			20	50.0	20	50.0		Ref.			10	25.0	30	75.0		Ref.		
Knowledge regarding “new normal” guidelines																								
More accurate	0	0.0	264	52.2	-	-	-	-	101	38.3	163	61.7	1.851	1.279	0.897–1.824	0.174	44	16.7	220	83.3	2.232	1.402	0.899–2.187	0.136
Less accurate	242	47.8	0	0.0	-	-			107	44.2	135	55.8	0.174	Ref.			53	21.9	189	78.1	(0.136)	Ref.		
Attitudes regarding “new normal” guidelines																								
More positive	135	45.3	163	54.7	1.851	1.279	0.897–1.824	0.174	0	0.00	298	58.9	-	-	-	-	40	13.4	258	86.6	15.453	**2.435**	**1.550–3.824**	**<** **0.001**
Less positive	107	51.4	101	48.6	(0.174)	Ref.			208	41.1	0	0.00	-	-			57	27.4	151	72.6	**(<0.001)**	Ref.		
Practices regarding “new normal” guidelines																								
More frequent	189	46.2	220	53.8	2.232	1.402	0.899–2.187	0.136	151	36.9	258	63.1	15.453	**2.435**	**1.550–3.824**	**<0.001**	0	0.00	409	80.8	-	-	-	-
Less frequent	53	54.6	44	45.4	(0.136)	Ref.			57	58.8	40	41.2	**(<0.001)**	Ref.			97	19.2	0	0.00	*-*	-		
Quality of life																								
High quality of life	125	45.1	152	54.9	1.788	1.270	0.894–1.804	0.181	93	33.6	184	66.4	14.346	**1.996**	**1.393–2.859**	**<0.001**	39	14.1	238	85.9	10.236	**2.070**	**1.319–3.249**	**0.002**
Low quality of life	117	51.1	112	48.9	(0.181)	Ref.			115	50.2	114	49.8	**(<0.001)**	Ref.			58	25.3	171	74.7	**(0.002)**	Ref.		

*Bold values are statistically significant*.

The binary logistic regression results revealed that the factors related to more accurate knowledge were education level [lower education vs. higher education: odds ratio (OR) = 0.27; 95% CI = 0.14–0.52, *p* < 0.001], government employee vs. unemployed OR = 2.57; 95% CI = 1.20–5.49, *p* = 0.015), monthly family income lower than 10,000 THB or 10,000–30,000 THB vs. monthly family income of more than 30,000 THB (OR = 0.34; 95% CI = 0.18–0.65, *p* = 0.001 and OR = 0.40; 95% CI = 0.26–0.62, *p* < 0.001, respectively), and never or sometimes used social media vs. used social media every day (OR = 0.54; 95% CI = 0.32–0.93, *p* = 0.026) (see Table 7). However, the multiple logistic regression results revealed that there were no significant differences between these factors and knowledge regarding “new normal” guidelines.

The findings for factors related to attitudes regarding “new normal” guidelines revealed that participants with more positive attitudes were significantly more likely to be (i) participants (62.3%) aged ≥ 60 years old compared with those (43.5%) aged between 20 and 24 years old (*p* < 0.001), (ii) participants (66.0%) who were divorced or separated compared with those (51.2%) who were single had less positive attitudes (*p* = 0.016), (iv) participants (63.9%) who were unemployed compared with those (43.7%) who were students (*p* < 0.001), (v)participants (63.6%) who never drank alcohol compared with those (50.8%) with a history of drinking alcohol within 6 months (*p* = 0.016), (vi) participants (63.1%) who had more frequent COVID-19 prevention practices compared with those (41.2%) with less frequent prevention practices (*p* < 0.001), (vii) participants (66.4%) with high QOL compared with those (49.8%) with low QOL (*p* < 0.001) (see Table 7).

The binary logistic regression results revealed that the factors related to more positive attitudes were age (those aged between 20 and 24 years vs. those aged ≥ 60 years old: OR = 0.47; 95% CI = 0.24–0.90, *p* = 0.024), students vs. unemployed (OR = 0.44; 95% CI = 0.20–0.96, *p* = 0.039), those with a history of drinking alcohol vs. those who did not drink alcohol within the last 6 months (OR = 0.59; 95% CI = 0.41–0.86, *p* = 0.005), more frequent practices vs. less frequent practices (OR = 2.44; 95% CI = 1.55–3.82, *p* < 0.001), and high QOL vs. low QOL (OR = 1.99; 95% CI = 1.39–2.86, *p* < 0.001) (see Table 7). Additionally, the multiple logistic regression results revealed that the factors associated with attitudes regarding “new normal” guidelines included drinking alcohol in the last 6 months, practice scores regarding “new normal” guidelines and QOL during the COVID-19 outbreak.

The findings of factors related to practices regarding “new normal” guidelines revealed that participants with more frequent practices were significantly more likely to be (i) participants (76.1%) who were single compared with those (82.0) who were divorced or separated (*p* = 0.035), (ii) participants (86.6%) who had more positive attitudes compared with those (85.9%) with less positive attitudes (*p* < 0.001), (iii) participants (85.9%) with a high QOL compared with those (74.7%) with a low QOL (*p* < 0.001) (see Table 7).

The results of binary logistic regression revealed that the factors related to more frequent practices were attitudes (more positive attitude vs. less positive attitude: OR = 2.44; 95% CI = 1.55–3.82, *p* < 0.001), high QOL vs. low QOL (OR = 2.07; 95% CI = 1.39–3.25, *p* = 0.002) (see Table 7). In addition, the results of multiple logistic regression revealed that the factors associated with practices regarding “new normal” guidelines included attitudes regarding “new normal” guidelines and QOL during the COVID-19 outbreak.

### Factors Related to Quality of Life During the COVID-19 Outbreak

The findings regarding factors related to QOL during the COVID-19 outbreak revealed that participants with high QOL were significantly more likely to be (i) participants (69.8%) aged ≥ 60 years old compared with (41.9%) aged between 20 and 24 years old (*p* = 0.001) (see Table 8), (ii)participants (54.0%) who were divorced or separated compared with those (48.3%) who were single (*p* = 0.016), (iii) participants (64.2%) with a higher level of education (above bachelor's degree) compare with those (63.8%) who had a lower level of education (below bachelor's degree) (*p* = 0.001), (iv) participants (58.3%) who were unemployed compare with those (39.8%) who were students (*p* = 0.001), (v) participants (61.7%) with more positive attitudes compared with those (44.7%) with less positive attitudes (*p* < 0.001), (vi) participants (58.2%) with more frequent prevention practices compared with those (40.2%) with less frequent practices (*p* = 0.002).

**Table 8:** Factors related to quality of life during the COVID-19 outbreak.

**Variables**	**Quality of life during the COVID-19 outbreak**								
	**Low quality of life**		**High quality of life**		***x* ^2^**	***p-value***	**OR**	**95% CI**	***p-value***
	***N***	**%**	***N***	**%**					
Gender									
Male	41	41.0	59	59.0	0.912	0.340	1.241	0.796–1.934	0.340
Female	188	46.3	218	53.7			Ref.		
Age (years)									
20–24	72	58.1	52	41.9	13.832	**0.001**	**0.312**	**0.157–0.620**	**0.001**
25–59	141	42.9	188	57.1			0.577	0.308–1.078	0.085
≥60	16	30.2	37	69.8			Ref.		
Marital status									
Unmarried	121	51.7	113	48.3	8.295	**0.016**	0.796	0.431–1.468	0.464
Married	85	38.3	137	61.7			1.373	0.740–2.548	0.315
Divorced	23	46.0	27	54.0			Ref.		
Education									
Lower education (below bachelor's degree)	64	36.2	113	63.8	14.313	**0.001**	0.987	0.520–1.871	0.967
Bachelor's degree	146	52.9	130	47.1			**0.498**	**0.271–0.915**	**0.025**
Higher education (above bachelor's degree)	19	35.8	34	64.2			Ref.		
Occupation									
Student	62	60.2	41	39.8	17.141	**0.001**	0.472	0.218–1.021	0.057
Government employee	40	32.8	82	67.2			1.464	0.683–3.140	0.327
Non-government employee	112	45.7	133	54.3			0.848	0.418–1.723	0.649
Unemployed	15	41.7	21	58.3			Ref.		
Monthly family income									
<10,000 THB	27	45.0	33	55.0	2.908	0.234	0.778	0.418–1.449	0.428
10,000–30,000 THB	153	47.8	167	52.2			0.659	0.456–1.057	0.089
>30,000 THB	49	38.9	77	61.1			Ref.		
Social media use									
Never or sometimes	22	34.4	42	65.6	3.502	0.063	1.682	0.972–2.911	0.063
Every day	207	46.8	235	53.2			Ref.		
Underlying disease									
Yes	56	45.5	67	54.5	0.005	0.945	0.986	0.655–1.482	0.945
No	173	45.2	210	54.8			Ref.		
Smoking									
Yes	12	48.0	13	52.0	0.080	0.778	0.890	0.398–1.992	0.778
No	217	45.1	264	54.9			Ref.		
Drinking alcohol									
Yes	92	49.7	93	50.3	2.355	0.125	0.753	0.523–1.082	0.125
No	137	42.7	184	57.3			Ref.		
Willingness to get vaccinated against COVID-19									
Yes	200	44.0	255	56.0	3.343	0.188	1.558	0.814–2.985	0.181
No	7	63.6	4	36.4			0.698	0.176–2.769	0.610
Not sure	22	55.0	18	45.0			Ref.		
Attitudes regarding “new normal” guidelines									
More positive	114	38.3	184	61.7	14.346	**<0.001**	**1.996**	**1.393–2.859**	**<0.001**
Less positive	115	55.3	93	44.7			Ref.		
Practices regarding “new normal” guidelines									
More frequent	171	41.8	238	58.2	10.236	**0.002**	**2.070**	**1.319–3.249**	**0.002**
Less frequent	58	59.8	39	40.2			Ref.		

*Bold values are statistically significant*.

The results of binary logistic regression revealed that the factors related to high QOL were age (those aged between 20 and 24 years old vs. those aged ≥ 60 years old: OR = 0.31; 95% CI = 0.16–0.62, *p* = 0.001), bachelor's degree vs. higher education (above bachelor's degree) (OR = 0.50; 95% CI = 0.27–0.92, *p* = 0.025), more positive attitudes vs. less positive attitudes (OR = 1.99; 95% CI = 1.39–2.86, *p* < 0.001), and more frequent practices vs less frequent practices (OR = 2.07; 95% CI = 1.32–3.25, *p* = 0.002) (see Table 8). In addition, the results of multiple regression revealed that the factors associated with QOL during the COVID-19 outbreak included attitudes and practices regarding “new normal” guidelines.

## Discussion

The current study was conducted to assess KAP regarding “new normal” guidelines and QOL among Thai people during the COVID-19 outbreak and to identify the predictors between KAP and QOL. The findings will be useful for developing health education programs and improving QOL. The findings revealed that approximately 16.6% of participants experienced job loss during COVID-19 curfew restrictions, which is a greater proportion than that reported in previous studies before travel restrictions and curfews were implemented (11.4%, from 21 April to 4 May 2020) (35) and during travel restrictions in May 2020 (36). The results indicated that monthly family income was decreased during travel restrictions and curfews among for 68.6% of people in Public Health Region 6, which was a similar proportion to that found in a previous study (36) and a greater proportion than that before curfew (35). The findings revealed that 87.4% of participants used online social media every day. Sheldon et al. reported that Thai people spent an average of 4.16 ± 2.60 h (SD) using online social media (37), and >60% spent more than 1 h per day using social media during the COVID-19 outbreak (38). Pan-ngum et al. reported that 88% of respondents indicated that they received COVID-19 information *via* social media or messenger apps (36). Tiaprapong et al. found that 12.0% of health science students smoked and 29.0% drank alcohol during the early implementation of “new normal” guidelines in September 2020 (28). The corresponding proportions in the current study were lower than those reported in this previous study, indicating that only 4.9% of participants smoked, while 36.6% drank alcohol during travel restrictions and curfews. Our findings indicated that 89.9% of participants expressed willingness to get vaccinated against COVID-19, whereas Kitro et al. found that less than half of Thai people and expatriates living in Thailand were willing to get vaccinated against COVID-19 (41.8%) (39). A series of mobile health applications have been introduced by the healthcare sector and implemented to aid disease control monitoring and prevention of widespread outbreaks (14–16). However, the study showed that the use of those applications was relatively rare because of the low rate of digital literacy among Thai people (40). The current study differs from previous studies regarding the use of online applications, such as ThaiChana and MorChana, for disease control and prevention, because some participants were confused about how to access the applications or did not understand the benefits of the applications.

The findings revealed that participants generally had accurate knowledge regarding “new normal” guidelines. The findings were similar to those of previous studies conducted among health science students, with scores ranging from 8 to 10 points (28), and Thai travelers, with an average score of 8.34 ± 12 points (SD) (41). Most respondents reported accurate knowledge regarding “new normal” guidelines: 97.6% of participants agreed that “People in contact with someone infected with SARS-CoV-2 should be immediately quarantined and tested for the virus,” which was similar to the findings of a previous study in China (20). Our results indicated that participants' knowledge was positively related to their attitudes, similar to the findings of a previous study (42). The current results also revealed that knowledge was positively related to practices, similar to previous studies conducted by Kamate et al. (43) and Al Ahdab et al. (44). In contrast, Prapaso et al. found a negative correlation between these factors (41). Furthermore, participants in the current study who had a lower level of education (below bachelor's degree) had less knowledge than those with a higher level of education (above bachelor's degree), which was similar to the findings of a previous study by Prapaso et al. (41). The findings revealed that participants who worked in the government sector had more accurate knowledge than those who were unemployed. As in the current study, Ferdous et al. found that occupation was associated with participants' level of knowledge (45). Participants who had a monthly family income lower than 10,000 THB or 10,000–30,000 THB had less accurate knowledge than those with a monthly family income higher than 30,000 THB. Similarly, Al-Wutayd et al. found more accurate knowledge among people with higher incomes (46). The current findings indicated that social media users had more accurate knowledge than those who never used social media. Puspitasari et al. reported that people who used social media frequently had more accurate knowledge (47). However, we found no significant differences between these factors and knowledge regarding “new normal” guidelines in multiple logistic regression analyses.

The current findings revealed that participants generally had positive attitudes regarding “new normal” guidelines, similar to previous reports (28, 41). We found that 82.6% of participants agreed that “It's important to maintain a distance of at least 2 meters to prevent the spread of COVID-19,” which is similar to the findings of a previous study in Bangladesh (48). Our results revealed that participants' attitudes were positively related to practices, similar to a previous study (44). Attitudes were positively related to QOL, as reported in a previous study (28). The present study revealed that participants aged between 20 and 24 years old had less positive attitudes than those aged ≥60 years old. Ferdous et al. also found that older people had more positive attitudes than younger people (45). The current results also revealed that single people had less positive attitudes than those who were divorced or separated, whereas Ferdous et al. reported that single people had more positive attitudes than those with other marital status (45). Pan-ngum et al. reported that mental health and wellbeing problems among single people were greater than those among people who lived with a partner and/or children/relatives (36). The present study revealed that participants who were students had less positive attitudes than participants who were unemployed, in accord with a previous study by Ferdous et al. (45). The current results revealed that participants who drank alcohol within the last 6 months had less positive attitudes than those who had never drunk alcohol, which was consistent with a previous study (49). The present study revealed that participants who had more frequent prevention practices had more positive attitudes than those with less frequent practices, consistent with a previous study conducted in Bangladesh (45). Moreover, participants with high QOL had more positive attitudes than those with low QOL, which was consistent with a previous study (28). Finally, multiple logistic regression revealed that the factors associated with attitudes regarding “new normal” guidelines included drinking alcohol within the last 6 months, practice scores regarding “new normal” guidelines, and QOL during the COVID-19 outbreak.

The findings revealed that many participants frequently engaged in practices regarding the “new normal” guidelines, which was similar to previous findings (28, 36, 41). In addition, 95.5% of participants always wore masks when they went to public places. The findings were similar to those of a study conducted among Thai travelers (41). The results revealed that participants who were single engaged in prevention practices less frequently than those who were divorced or separated, consistent with a study in Bangladesh (45). Our study revealed that participants who had more positive attitudes engaged in prevention practices more frequently than those with less positive attitudes. This result was consistent with the findings of a previous study (28). Moreover, the present findings indicated that participants who had a high QOL engaged in more frequent practices to prevent COVID-19 than those with low QOL. Additionally, the present multiple logistic regression results revealed that factors associated with practices regarding “new normal” guidelines included attitudes regarding “new normal” guidelines and QOL during the COVID-19 outbreak.

The current results revealed that 54.7% of participants had high QOL scores, ranging from 96 to 130 points. This finding was similar to the results of a previous study conducted among health science students (28). The current study revealed that participants aged between 20 and 24 years old had lower QOL than those aged ≥60 years old. Algahtani et al. reported that physical and mental changes among older people were associated with QOL (50). In addition, we found that participants who were single had lower QOL than those who were divorced or separated. Woon et al. also found that divorced people had a relatively high level of QOL (51). The present study revealed that the participants with a lower level of education (below bachelor's degree) had lower QOL than those with a higher level of education (above bachelor's degree), in accord with a previous study in Italy (52). The current study revealed that participants who were students had a lower QOL than those who were unemployed, consistent with a previous study in Saudi Arabia (50). Moreover, the current multiple logistic regression results indicated that various factors were associated with QOL during the COVID-19 outbreak, including attitudes and practices regarding the “new normal.”

### Limitations

The current study involved several limitations. First, this study used an online-based cross-sectional survey method to avoid possible COVID-19 transmission. Therefore, causal relationships could not be assessed, and the sample was restricted to individuals with Internet access. Thus, the current findings are unlikely to represent an accurate reflection of the whole population in Public Health Region 6. Second, most participants were women, and more than half held bachelor's degrees. Thus, the results might not be representative of male participants with an education level below a bachelor's degree. A previous study reported that women had significantly larger social networks than men (53). Moreover, a study in Colombia reported that people with higher levels of education used social media more than those with lower levels of education (54). However, there were no significant differences in attitudes and practices by gender or education level. Thus, a future study should collect more comprehensive data on gender and academic levels. The present study used the KAP approach to determine the association between KA, QOL, and practices regarding “new normal” guidelines, however further research is needed to explore the Ajzen's approach (30) to find the relevant beliefs that should be targeted in an intervention.

## Conclusion

Our findings indicate that Thai people and policymakers should emphasize KAP in the use of mobile health applications for contact tracing and vaccination services. Enhancement of attitudes and QOL is also important for improving practices in the general population during the COVID-19 pandemic. Factors related to KAP are crucial for developing prevention and control programs to mitigate the spread of the COVID-19 outbreak.

## Data Availability Statement

The raw data supporting the conclusions of this article will be made available by the authors, without undue reservation.

## Ethics Statement

The studies involving human participants were reviewed and approved by the Human Research Ethics Committee Chulabhorn Research Institute. Formal ethics approval was granted on 23 April 2021 (Project Code: 025/2564). The objectives and procedures of the study were described in the consent form. Anonymity and confidentially were strictly maintained. The patients/participants provided their written informed consent to participate in this study.

## Author Contributions

PW and WM: conceptualization, writing—original draft preparation, and methodology. WP, SS, and BB: supervision. WM: visualization and formal analysis. PW, WM, WP, SS, and BB: writing—review and editing. All authors have read and approved the final version of the manuscript.

## Funding

This research project is funded by the Chulabhorn Royal Academy. This study was partially funded by the National Research Council of Thailand (NRCT) and the Wellcome Trust (Grant number 220211).

## Conflict of Interest

The authors declare that the research was conducted in the absence of any commercial or financial relationships that could be construed as a potential conflict of interest.

## Publisher's Note

All claims expressed in this article are solely those of the authors and do not necessarily represent those of their affiliated organizations, or those of the publisher, the editors and the reviewers. Any product that may be evaluated in this article, or claim that may be made by its manufacturer, is not guaranteed or endorsed by the publisher.
